# Correction to Bindemann et al. (2022)

**DOI:** 10.1037/mac0000050

**Published:** 2022-09-29

The article “Face Identification in the Laboratory and in Virtual Worlds,” by Markus Bindemann, Matthew C. Fysh, Iliyana V. Trifonova, John Allen, Cade McCall, and A. Mike Burton (*Journal of Applied Research in Memory and Cognition*, 2022, Vol. 11, No. 1, pp. 120–134, https://doi.org/10.1016/j.jarmac.2021.07.010), should have been published under the terms of the Creative Commons Attribution License (CC BY 3.0). Therefore, the article was amended to list the authors as copyright holders, and information about the terms of the CC BY 3.0 was added to the author note. In addition, the article is now open access. The online version of this article has been corrected.

